# Case report: Percutaneous adrenal arterial embolization cures resistant hypertension

**DOI:** 10.3389/fcvm.2022.1013426

**Published:** 2022-10-11

**Authors:** Yaqiong Zhou, Dan Wang, Qiting Liu, Jixin Hou, Peijian Wang

**Affiliations:** ^1^Department of Cardiology, Clinical Medical College and The First Affiliated Hospital of Chengdu Medical College, Chengdu, China; ^2^Key Laboratory of Aging and Vascular Homeostasis of Sichuan Higher Education Institutes, Chengdu, China

**Keywords:** primary aldosteronism, hypertension, adrenal arterial embolization, ablation, case report

## Abstract

**Background:**

Primary aldosteronism is a common cause of resistant hypertension. Patients with primary aldosteronism due to aldosterone-producing adenoma are generally treated with unilateral adrenalectomy or medical therapy. Superselective adrenal arterial embolization is an alternative treatment for patients with unilateral primary aldosteronism.

**Case summary:**

We present a 39-year-old male patient with a 5-year history of primary aldosteronism and secondary hypertension. The patient refused adrenalectomy while accepted pharmacotherapy. Despite taking adequate dose of spironolactone, the patient experienced repeatedly muscle weakness due to hypokalemia and had poor blood pressure control with left ventricular hypertrophy and renal dysfunction. Aldosterone-producing adenoma in the left adrenal gland was confirmed by computerized tomography and adrenal venous sampling. The left middle adrenal artery, which was confirmed to provide the main arterial supply to the aldosterone-producing adenoma, was embolized by injecting 2 ml ethanol. The embolization normalized his blood pressure for up to 3 months and reversed left ventricular hypertrophy.

**Conclusion:**

Superselective adrenal arterial embolization could be an alternative treatment for patients with aldosterone-producing adenoma who refuse adrenalectomy.

## Introduction

Primary aldosteronism is a common cause of secondary hypertension and is associated with a higher risk of cardiovascular and renal complications than essential hypertension (1). Aldosterone-producing adenoma, also known as aldosteronoma, is a major cause of primary aldosteronism and is characterized by autonomous aldosterone secretion (2). According to current clinical practice guidelines, unilateral laparoscopic adrenalectomy is the preferred treatment for aldosterone-producing adenoma in patients with primary aldosteronism (3–5). However, unilateral adrenalectomy can only cure hypertension in about half of patients with aldosterone-producing adenoma (6) based on the Primary Aldosteronism Surgical Outcome criteria (7). Patients who do not receive surgery are usually treated with mineralocorticoid receptor antagonists, such as spironolactone (3). A large case-control study demonstrated that, despite treated with unilateral adrenalectomy or mineralocorticoid receptor antagonists, patients with primary aldosteronism had increased cardiovascular mortality when compared with patients with essential hypertension (8). Recently, superselective adrenal arterial embolization has been used as an alternative treatment for patients with unilateral aldosterone-producing adenoma (9), but its long-term effectiveness and safety warrant further investigation. In this report, we present a case with aldosterone-producing adenoma and secondary hypertension that was resistant to treatment of mineralocorticoid receptor antagonists. The patient refused either open or laparoscopic adrenalectomy. Fortunately, his hypertension was cured and cardiac hypertrophy was reversed by superselective adrenal arterial embolization.

## Case presentation

**FIGURE 1:**
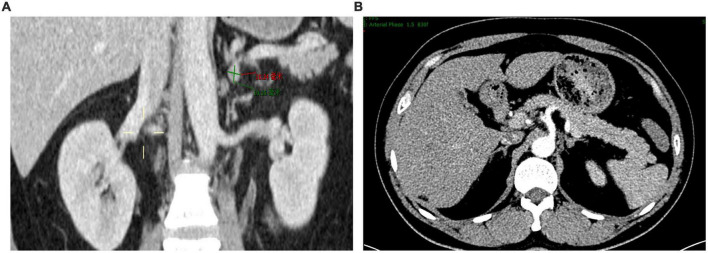
Contrast-enhanced computerized tomography scan showing a small left adrenal adenoma. **(A)** Coronal view. **(B)** Cross-sectional view.

We present a 39-year-old male patient with a 5-year history of chronic hypertension. His highest blood pressure record was 198/118 mmHg. One year ago, the patient had severe fatigue and muscle weakness due to hypokalemia (2.6 mmol/L). He was diagnosed with primary aldosteronism in a local hospital based on elevated serum aldosterone (515.0 pg/ml) and suppressed renin (4.5 pg/ml). The patient was treated with spironolactone (40 mg twice daily), but his blood pressure was poorly controlled, ranging from 140/90 to 180/110 mmHg. Two days before admission, the patient developed severe muscle weakness with cramps and impaired physical mobility. At admission, his blood pressure was 176/106 mmHg. Medication adherence was confirmed by structured interviews. His plasma potassium level was 1.8 mmol/L, and 24-h urinary potassium excretion was 150 mmol. Hypokalemia was corrected with intravenous administration of potassium. Further laboratory works showed that plasma concentrations of aldosterone and renin in supine position were 808.6 and 1.4 pg/ml, respectively, while were 461.1 and 10.8 pg/ml after standing. Aldosterone to renin ratios were 565 in supine position and 43 after standing. The result of salt loading test was positive. The microalbumin-to-creatinine ratio was increased at 418 mg/g. The 24-h urine vanillylmandelic acid and plasma cortisol rhythm were within normal ranges. Transthoracic echocardiograph showed enlarged left atrium (35 mm), increased left ventricular end-diastolic volume (158 ml), and increased left ventricular mass (160 g/m^2^) with normal left ventricular ejection fraction (74%). The contrast-enhanced computerized tomography scan of the abdomen revealed a small left adrenal adenoma of 1.0 × 0.8 cm (Figures 1A,B). The left and right adrenal veins were sequentially sampled without cosyntropin stimulation using a 5-french Tiger catheter (Terumo, Tokyo, Japan) as previously described (10). The aldosterone levels in the left and right adrenal vein samples were 63622 and 9294 pg/ml, while the aldosterone level in the inferior vena cava sample was 720 pg/ml. The cortisol levels in the left and right adrenal vein samples were 18732 and 18586 nmol/L, while the cortisol level in the inferior vena cava sample was 308 nmol/L. The aldosterone-to-cortisol concentration ratio was 3.40 and 0.50 for the left and right sides, respectively. The calculated lateralization index for the left side as the dominant side was 6.8, and the contralateral suppression index was 0.21, confirming the left adrenal aldosterone-producing adenoma. The patient was diagnosed with primary aldosteronism due to aldosterone-producing adenoma and secondary hypertension with hypertensive heart disease and renal dysfunction. Considering that treatment with spironolactone was ineffective in the patient, left adrenalectomy was recommended. However, the patient refused open or laparoscopic surgery. Therefore, we arranged an interventional procedure of superselective adrenal arterial embolization.

**FIGURE 2:**
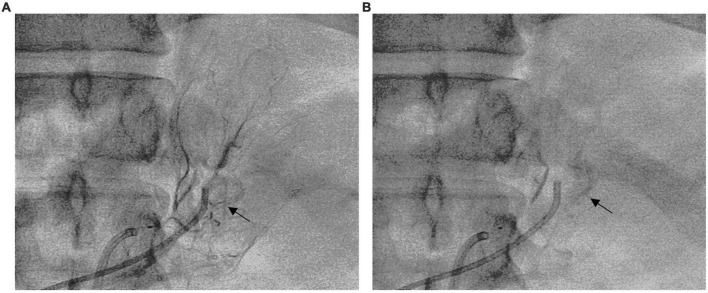
Superselective adrenal arterial embolization. **(A)** Angiography of the left middle adrenal artery showing enhancement of the left adrenal adenoma (Arrow). **(B)** Post-embolization angiography of the left middle adrenal artery confirming no blood flow to the adenoma (Arrow).

**FIGURE 3:**
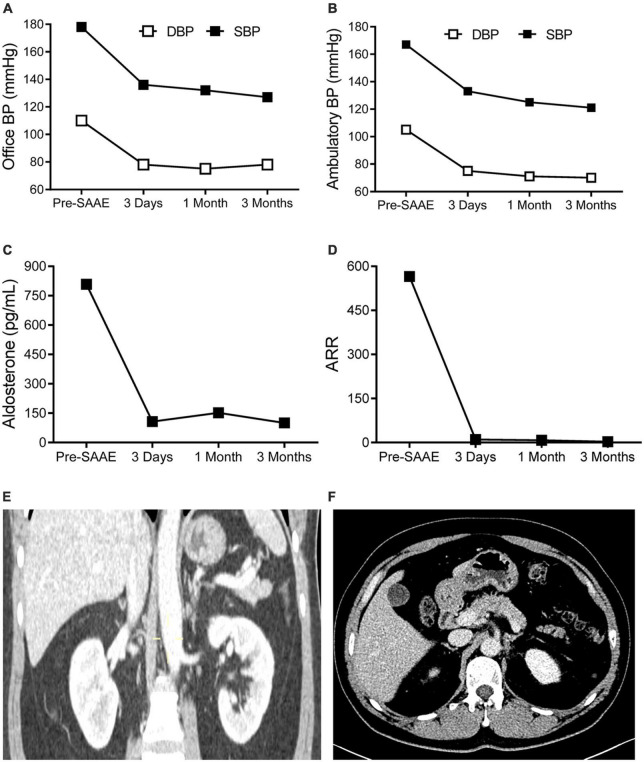
The effects of superselective adrenal arterial embolization (SAAE). Office **(A)** and mean ambulatory blood pressure (BP) **(B)**, plasma aldosterone levels **(C)**, and aldosterone to renin ratios (ARR) **(D)** of the patient before SAAE and 3 days, 1 month, and 3 months after SAAE. Contrast-enhanced computerized tomography showing disappearance of the left adrenal adenoma. **(E)** Coronal view. **(F)** Cross-sectional view. Abbreviations: DBP, diastolic blood pressure; SBP, systolic blood pressure.

The left superselective adrenal arterial embolization was performed with a 6-french Judkins right (JR 3.5) catheter (Launcher, Medtronic, Minnesota, USA). The arterial supply to the aldosterone-producing adenoma was confirmed as the left middle adrenal artery arising from the abdominal aorta by angiography (Figure 2A). The left middle adrenal artery was embolized by injecting 2 ml ethanol. Angiography at 5 min post-embolization confirmed no blood flow to the aldosterone-producing adenoma (Figure 2B). The left superselective adrenal arterial embolization succeed without hypertensive crisis or complications during or after the procedure. All antihypertensive medications were discontinued immediately after the embolization procedure. His office blood pressure decreased to 136/78 mmHg on day 3 post-embolization and was kept below 140/80 mmHg for up to 3 months after the procedure (Figure 3A). Ambulatory blood pressure monitoring confirmed that hypertension of the patient was cured by the left superselective adrenal arterial embolization (Figure 3B). Plasma aldosterone and aldosterone-to-renin ratio were corrected on day 3 post-embolization and maintained at low levels for 3 months (Figures 3C,D). Plasma potassium levels on day 3, 1 month, and 3 months after the procedure were 4.62, 4.33, and 4.52 mmol/L, respectively. Abdominal computerized tomography scan at 3 months post-procedure demonstrated disappearance of left adrenal adenoma (Figures 3E,F). Moreover, cardiac hypertrophy was reversed with left ventricular mass of 105 g/m^2^ measured by echocardiography at 3 months post-procedure without pharmacological treatment.

## Discussion

In this report, we described a patient with primary aldosteronism due to aldosterone-producing adenoma and hypertensive heart disease. The patient’s hypertension was resistant to pharmacological treatment with spironolactone while was cured by superselective adrenal arterial embolization.

Primary aldosteronism is found to be a more common cause of secondary hypertension than previously reported (11). Rather than a benign form of moderate hypertension, secondary hypertension caused by primary aldosteronism is associated with worse cardiovascular and renal consequences (12). Moreover, cardiovascular complications were the main cause of death in patients with primary aldosteronism and were more frequent in patients with primary aldosteronism than in essential hypertensive controls (8). Excessive aldosterone secretion in primary aldosteronism not only causes hypertension but also induces cardiac fibrosis, left ventricular hypertrophy, and diastolic heart failure independent of blood pressure elevation (13). Unilateral primary aldosteronism due to adrenal adenoma or hyperplasia is mainly treated by adrenalectomy or alternatively controlled by medical treatment with mineralocorticoid receptor antagonists (14). However, a considerable proportion of patients still requires antihypertensive medications after unilateral adrenalectomy (6). This calls for earlier and more effective treatment to improve the prognosis of patients with primary aldosteronism.

Unilateral laparoscopic adrenalectomy is recommended over medical treatment for patients with unilateral primary aldosteronism according to the current clinical practice guideline (3). Real word studies demonstrated that adrenalectomy was only performed in 87.6% of patients with unilateral primary aldosteronism confirmed by adrenal vein sampling and was even lower (78.2%) in Asian populations (15). Many factors, including advanced age and comorbidities, contributed to patients’ decision of not undergoing adrenalectomy (15). Patients who are unable or unwilling to undergo adrenalectomy are generally treated with mineralocorticoid receptor antagonists. Evidence indicates that mineralocorticoid receptor antagonists and adrenalectomy achieve similar blood pressure reduction (16). However, a real word investigation demonstrated that treatment with mineralocorticoid receptor antagonists failed to revert renin suppression in 43% of patients with primary aldosteronism (17). Failure in renin suppression was associated with enhanced cardiovascular and renal mortality in these patients. Moreover, 30.7% of treated patients suffered adverse events from spironolactone (17). A recent study demonstrated that mineralocorticoid receptor antagonists-induced renin suppression was strongly time- and dose-dependent (18). Our patient described in the present report refused adrenalectomy and was refractory to spironolactone treatment, resulting in the development of cardiovascular and renal complications. Fortunately, superselective adrenal arterial embolization cured his hypertension and reversed myocardial remodeling.

Superselective adrenal arterial embolization was reevaluated recently in primary aldosteronism. In a recent randomized controlled trial, they compared the effectiveness of adrenal arterial embolization and medical treatment and found that the two treatments achieved comparable blood pressure reduction (9). However, the long-term cardiovascular benefits of adrenal arterial embolization need further investigation (9). In the present report, superselective adrenal arterial embolization provided cardiovascular benefits in addition to blood pressure normalization. Based on this interesting finding, it is worth testing the effects of superselective adrenal arterial embolization in more patients with unilateral primary aldosteronism.

In conclusion, superselective adrenal arterial embolization could be an alternative treatment for patients with aldosterone-producing adenoma who refuse adrenalectomy.

## Data availability statement

The original contributions presented in this study are included in the article/supplementary material, further inquiries can be directed to the corresponding author.

## Ethics statement

The studies involving human participants were reviewed and approved by the Institutional Review Board of Chengdu Medical College. The patients/participants provided their written informed consent for the publication of this case report.

## Author contributions

PW drafted and revised the manuscript. All authors performed the embolization procedure, collected data, read, and approved the final manuscript.
